# Diagnostic Accuracy of Preoperative Magnetic Resonance Imaging for Staging Endometrial Carcinoma: A Retrospective Single-Centre Study

**DOI:** 10.7759/cureus.116394

**Published:** 2026-09-16

**Authors:** Arun Sanap, Sachin R Chaudhari, Ashwini Umredkar, Anita Yadav, Medha Davile, Anusha Kamath

**Affiliations:** 1 Obstetrics and Gynaecology, All India Institute of Medical Sciences, Nagpur, Nagpur, IND; 2 Pathology, All India Institute of Medical Sciences, Nagpur, Nagpur, IND; 3 Radio-diagnosis, All India Institute of Medical Sciences, Nagpur, Nagpur, IND

**Keywords:** diagnostic accuracy, diagnostic efficacy, endometrial ca, endometrial carcinoma, gynaecologic oncology, histopathology (hp), radiology

## Abstract

Background

Globally, endometrial cancer is becoming more common. However, reporting has been delayed till the very end in India. Pre-operative mapping of the disease's extent, particularly myometrial invasion, aids in surgical planning and the selection of adjuvant therapy, lymphadenectomy, and fertility preservation. Because of its higher tissue resolution, magnetic resonance imaging (MRI) is the recommended non-invasive staging method; nonetheless, reported concordance with histology varies greatly. This study investigates the accuracy with which MRI depicts the actual disease stage in practical settings.

Methodology

We retrospectively reviewed 32 consecutive patients with primary endometrial carcinoma who underwent preoperative MRI followed by definitive surgical staging (January 2022-December 2023, tertiary academic centre). Socio-demographic, clinical, and imaging data were reviewed, and MRI findings for myometrial, cervical, adnexal, and nodal involvement were compared with hysterectomy histopathology (gold standard). Statistical analysis generated accuracy, sensitivity, specificity, and predictive values for key parameters.

Results

Participants had a mean age of 57 years; 87.5% were postmenopausal, and 59.4% were obese. Postmenopausal bleeding was the presenting symptom in 87.5%. MRI accurately determined myometrial invasion depth in 62.5% of cases, with an overall staging concordance of 53.1%. Upstaging occurred in 21.9% and downstaging in 25%. For myometrial invasion, MRI showed sensitivity 58.3%, specificity 65.0%, Positive Predictive Value (PPV) 50.0%, and Negative Predictive Value (NPV) 72.2%. Discordance was most frequent in borderline (<50%) invasion cases, where subtle histologic features escaped radiologic detection.

Conclusion

MRI remains an important tool for preoperative risk stratification in endometrial carcinoma and helps in counselling and guiding surgical management. However, the diagnostic accuracy can be increased with the use of more refined imaging such as diffusion-weighted and contrast-enhanced sequences, and closer multidisciplinary interpretation. Strengthening MRI-pathology alignment can reduce unnecessary lymphadenectomies and better support fertility-sparing pathways, particularly in resource-limited settings.

## Introduction

In affluent countries, endometrial carcinoma is the most prevalent gynaecological malignancy [1-2]. However, in developing countries, it is the second most common gynaecological malignancy, with an incidence of 5.9 per 100,000 women. In India, the incidence is 4.3 per 100,000 women [3]. It is more common in the sixth and seventh decades of life, with a mean patient age of 65 years [4].

Endometrial cancer can be broadly divided into two types: Type I and Type II. Type 1 endometrial carcinoma, which is usually endometrioid, accounts for 75-80% of endometrial cancers and often arises on a background of atypical hyperplasia [5]. Type II is less oestrogen-dependent and shows a more aggressive behaviour with a tendency for deeper myometrial invasion. Serous, clear-cell, and undifferentiated carcinoma are common histological subtypes of Type II cancer [6].

Prognosis of endometrial cancer is related to stage at presentation, grade, lymph node involvement/lymphovascular space invasion (LVSI), and morphological subtype. Surgical staging is extremely important for its management, and complete surgical staging involves hysterectomy, bilateral salpingo-oophorectomy, pelvic/para-aortic lymphadenectomy, and omentectomy based on risk factors and tumour type [7]. Accurate disease diagnosis and extent, identification of patients at high risk for recurrence, patient customisation for adjuvant therapy that reduces relapses, and prognostic evaluation are all made possible by surgical staging [8].

The extent of lymphadenectomy for establishing prognostic and therapeutic benefit is controversial [9]. Nonetheless, one of the most significant prognostic markers for endometrial cancer is nodal involvement. Although clinical evaluation with diagnostic imaging cannot replace surgical staging, it enables preoperative planning of the surgical procedure and optimisation of the surgical procedure and a tailored therapeutic strategy. When a patient is unsuited for surgery, preoperative clinical and radiological staging is helpful in determining whether adjuvant therapy is necessary [10]. By classifying patients into high-risk and low-risk groups, it helps prevent needless lymphadenectomy in low-risk situations. When endometrial lesions are removed and hormone therapy is started, preoperative examination also aids in the management and follow-up of patients who wish to preserve their fertility [11-12].

This article was previously presented as an e-poster at the 2026 European Society of Gynaecological Oncology (ESGO) Congress on 26th February, 2026.

## Materials and methods

A retrospective cross-sectional study was conducted to assess the correlation between preoperative MRI findings and the histopathological findings in the hysterectomy specimens of all primary endometrial cancers. After obtaining Institutional Ethical Clearance (IEC; approval no. IEC/Pharmac/2024/182), all cases of carcinoma of the endometrium who underwent preoperative MRI and surgery at the Department of Obstetrics and Gynaecology at our centre from January 2022 to December 2023 were included in the study.

The sample size was based on convenience sampling. The present study included patients with histopathologically confirmed endometrial carcinoma who underwent preoperative MRI evaluation followed by surgical staging and histopathological assessment. Patients with recurrent disease, prior pelvic malignancy treatment, contraindications to MRI, or incomplete histopathological data were excluded from the analysis.

MRI interpretation was performed independently of the final histopathological findings, and the radiologist was blinded to the histopathological reference standard. MRI examinations were performed on a 3T Siemens MAGNETOM Skyra scanner (Siemens Healthineers, Erlangen, Germany) using a pelvic phased-array coil. The protocol includes T2-weighted multiplanar imaging, axial T1-weighted imaging, Diffusion-Weighted Imaging (DWI) with Apparent Diffusion Coefficient (ADC) maps, and dynamic contrast-enhanced T1-weighted imaging after gadolinium. Slice thickness was 3-4 mm. Images were independently reviewed by an experienced radiologist with 15 years of experience in reporting pelvic MRI scans. MRI interpretation was performed independently of the final histopathological findings, and the radiologist was blinded to the histopathological reference standard.

The data were obtained from retrospective review of medical case records. Age, parity, primary complaints (such as heavy menstrual bleeding or postmenopausal bleeding), medical and family histories (such as diabetes mellitus and hypertension), clinical examination results, and preoperative clinical staging (based on radiological findings (MRI), tumour grade, and endometrial biopsy histology) were all recorded.

The final histological diagnosis served as the basis for surgical staging. The accuracy of MRI in determining the depth of myometrial invasion was assessed by comparing MRI classification with final histopathology, with myometrial invasion categorized as <50% or ≥50%. Second, overall staging concordance was assessed by comparing the preoperative MRI-based International Federation of Gynecology and Obstetrics (FIGO) stage with the final histopathological FIGO stage. Overall staging was categorised as concordant, upstaged, or downstaged according to the comparison between the MRI-based and final histopathological stages.

A spreadsheet was created using the gathered data. Numerical variables were used to capture categorical and continuous data, which were then reported as mean, median, and frequency, respectively. IBM SPSS Statistics for Windows, Version 22 (Released 2013; IBM Corp., Armonk, New York, United States) was used to analyse the data.

## Results

The present study included 32 patients diagnosed with endometrial carcinoma, with a mean age of 57 years. The majority of them were >60 years old, married, parous with parity one or two, and postmenopausal. Most of them had comorbidities such as hypertension, diabetes, and obesity. Table 1 shows the socio-demographic characteristics of the study population.

**Table 1:** Distribution of the study subjects according to the demographic factors (N=32)

	Number of patients (n)	Percentage
Age in years:		
<50	10	31.25%
50-60	10	31.25%
>60	12	37.50%
Marital status: Unmarried	2	6.25%
Married	30	93.75%
Menopausal status: Pre	4	12.50%
Post	28	87.50%
Parity: Nulliparous	2	6.25%
1-2	16	50%
>/=3	14	43.75%
Comorbidity		
Hypertension	17	53.10%
Diabetes Mellitus	5	15.60%
Obesity	19	59.40%

The most common presentation among the patients was postmenopausal bleeding or blood-tinged discharge (28/32, 87.5%) and heavy menstrual bleeding (4/32, 12.5%). Pelvic examination showed 90.6% patients had uterine size of six to 12 weeks while only few 9.4% had uterine size greater than 12 weeks. No patient had any palpable abdominal mass and the parametrium and adnexa were free of disease. Endometrial thickness of >4 mm on ultrasound was present in all of them. 

MRI correctly predicted myometrial involvement when compared to histopathology in 20/32 (62.5%) cases. This is depicted in Table 2.

**Table 2:** Diagnostic performance of MRI for detection of myometrial invasion compared with histopathology TP: True positive; FN: False negative.

MRI assessment of myometrial invasion	Histopathology: Deep invasion ≥50%	Histopathology: No deep invasion <50%	Total
MRI positive (≥50% invasion)	7 (TP)	7 (FP)	14
MRI negative (<50% invasion)	5 (FN)	13 (TN)	18
Total	12	20	32

The clinical staging was concordant with histopathological staging in 53.1% (17/32) cases. Using histopathology as the reference standard, 12 of 32 patients had deep myometrial invasion (≥50%). MRI correctly identified seven of these 12 patients, while five were classified as having <50% invasion. Among the 20 patients without deep myometrial invasion on histopathology, MRI correctly classified 13 and incorrectly classified seven as having deep invasion. Upstaging was seen in 7/32 cases (21.9%), while downstaging occured in 8/32 (25.0%). Concordant staging was seen in 17/32 (53.1%) of the cases. 

In the evaluation of myometrial invasion in endometrial cancer, MRI demonstrated modest diagnostic performance. The overall accuracy was 62.5%, indicating correct classification of invasion depth in approximately two-thirds of cases. The sensitivity (58.3%; 95% CI 27.7-84.8%) reflects MRI’s ability to correctly identify cases with deep myometrial invasion (Table 3), whereas the specificity (65.0%; 95% CI 40.8-84.6%) indicates its capacity to correctly rule out deep invasion in patients with superficial involvement.

**Table 3:** Correlation of preoperative MRI staging with final histopathological staging

Parameter	Diagnostic performance
Accuracy	62.50%
Sensitivity	58.30%
Specificity	65%
Positive predictive value	50%
Negative predictive value	72.20%

There was only fair agreement between MRI and histopathology, in assessing depth of myometrial invasion (κ=0.23), indicating limited concordance. 

The positive predictive value (PPV) was 50.0% (95% CI 23.0-77.0%), suggesting that only half of the MRI-predicted deep invasion cases were confirmed on histopathology. Conversely, the negative predictive value (NPV) was 72.2% (95% CI 46.5-90.3%), demonstrating that MRI was more reliable in excluding deep myometrial invasion than in confirming it. The correct staging discordance values are 21.9% (7/32) for upstaging and 25.0% (8/32) for downstaging. The coefficient κ=0.23 has been explicitly interpreted as fair agreement between MRI and histopathology.This shows that MRI has limited positive concordance but reasonable negative agreement with histopathology. The diagnostic accuracy was 62.5% (95% CI 43.7-78.9%)

Overall, these findings indicated that while MRI provided useful preoperative information, its performance in accurately determining the depth of myometrial invasion remained limited.

## Discussion

Endometrial carcinoma requires accurate preoperative assessment to facilitate surgical planning, risk stratification, and selection of appropriate adjuvant treatment [13]. Although definitive staging remains dependent on surgical and histopathological assessment, preoperative imaging provides important information regarding the extent of disease, particularly myometrial invasion, cervical stromal involvement, adnexal disease, and lymph node involvement. MRI is an important imaging modality because of its superior soft-tissue contrast and ability to delineate the endometrial-myometrial interface and assess local tumour extension [14].

MRI is recommended by the European Society of Urogenital Radiology (ESUR) and the National Comprehensive Cancer Network (NCCN) for patients with stage IA illness, endometrial malignancies with serous histologic subtype, and possible cervical invasion [15]. MRI is also a useful method for assessing the degree of illness, especially the depth of myometrial invasion, cervical involvement, and lymph node metastases. The correlation between MRI findings and histopathological results obtained from surgical specimens is crucial for determining the reliability and clinical utility of MRI in endometrial cancer management [16]. 

In the present study, MRI correctly assessed the depth of myometrial invasion in 62.5% of cases, with a sensitivity of 58.3%, specificity of 65.0%, positive predictive value of 50.0%, and negative predictive value of 72.2%. Agreement between MRI and histopathology was only fair (κ=0.23), indicating limited concordance between the two modalities. These findings suggest that although MRI provides useful preoperative information, its ability to distinguish superficial from deep myometrial invasion was limited in this cohort. Staging discordance was observed in 46.9% of patients, with 21.9% being upstaged and 25.0% being downstaged following final histopathological assessment. Such discrepancies may occur because subtle or borderline myometrial invasion can be difficult to distinguish radiologically, particularly when the endometrial-myometrial interface is poorly defined or when coexisting adenomyosis or leiomyomas alter the normal uterine architecture.

Our findings are broadly within, although at the lower end of, the diagnostic performance reported in the international literature. Previous studies have reported MRI sensitivity of approximately 50-80% and specificity of 74-90% for detecting deep myometrial invasion [17,18]. Wu et al. and Dokter et al. also reported that MRI is valuable for preoperative staging but may underestimate or overestimate invasion in selected cases [13,14,18-20]. The comparatively lower accuracy observed in our study may be explained by the small sample size, differences in tumour characteristics, MRI protocols, radiologist experience, and the relatively small number of patients available for analysis. In particular, the wide confidence intervals around our diagnostic performance estimates indicate substantial uncertainty and require cautious interpretation.

Our results should also be considered in the context of contemporary international recommendations. The European Society of Gynaecological Oncology, the European Society for Radiotherapy and Oncology, and the European Society of Pathology (ESGO/ESTRO/ESP) guidelines recognize pelvic MRI and expert transvaginal ultrasound as appropriate imaging modalities for preoperative assessment of endometrial carcinoma, particularly for evaluation of myometrial and cervical stromal invasion [21].

A contemporary study evaluating MRI against histopathology reported an overall staging accuracy of 74%, with both underestimation and overestimation of myometrial invasion observed [22]. An MRI is particularly useful when a comprehensive assessment of local and extrauterine disease is required; however, imaging remains complementary to histopathological assessment rather than a replacement for definitive surgical staging. This is especially relevant in patients with borderline degrees of myometrial invasion, in whom the distinction between less than 50% and 50% or greater invasion may be difficult on imaging.

Recent evidence further supports a more nuanced interpretation of the role of MRI. A 2024 systematic review and meta-analysis involving 18 studies and 1,548 patients found pooled sensitivity and specificity for detection of deep myometrial invasion of 81.1% and 83.8%, respectively, for MRI, compared with 76.6% and 87.4% for transvaginal ultrasound. The differences between MRI and transvaginal ultrasound were not statistically significant, suggesting broadly comparable diagnostic performance [23]. Similarly, a 2023 head-to-head meta-analysis restricted to low-grade endometrioid endometrial carcinoma reported pooled MRI sensitivity and specificity of 65% and 85%, respectively, compared with 71% and 76% for transvaginal ultrasound, with no statistically significant difference between the modalities [24]. A further 2025 meta-analysis of 22 studies reported sensitivity and specificity of 79% and 84%, respectively, for MRI and 76% and 84% for transvaginal ultrasound, again demonstrating comparable diagnostic performance [25]. These findings emphasise that the clinical utility of preoperative imaging depends not only on the modality itself but also on operator expertise, imaging quality, patient characteristics, and local availability of resources.

The diagnostic performance of MRI is also influenced by the imaging protocol and the sequences used. Conventional T2-weighted imaging remains fundamental for assessment of the endometrial-myometrial interface, while DWI and dynamic contrast-enhanced sequences may provide additional information regarding tumour extent. Contemporary studies have evaluated newer diffusion-weighted techniques and have demonstrated their potential to improve image quality and assessment of myometrial invasion, although differences between diffusion-weighted and contrast-enhanced techniques are not consistently significant [26]. Therefore, optimization and standardization of MRI protocols, together with experienced radiological interpretation, may improve diagnostic reproducibility.

The importance of accurate assessment of myometrial invasion remains substantial, but its role should be interpreted in the context of the evolution of endometrial cancer staging. The 2023 FIGO staging system has incorporated histological characteristics, lymphovascular space invasion, and molecular classification in addition to anatomical disease extent [27]. In particular, the revised system recognizes distinct molecularly defined subgroups, including DNA polymerase epsilon (POLE)-mutated and p53-abnormal tumours, which may result in stage modification in selected early-stage cancers. Thus, while myometrial invasion remains an important component of anatomical assessment, contemporary risk stratification increasingly integrates imaging, histopathology, and molecular classification rather than relying on any single parameter [27].

From a clinical perspective, preoperative MRI remains valuable for providing an anatomical roadmap and assisting surgical planning. However, the relatively modest diagnostic performance observed in our cohort and the fair agreement with histopathology indicate that MRI findings should not be interpreted in isolation. This is particularly important when imaging suggests borderline myometrial invasion, where radiological overestimation may potentially influence surgical decision-making. In the contemporary era of sentinel lymph-node assessment and molecular risk stratification, imaging should be regarded as one component of a multidisciplinary preoperative assessment rather than as the sole determinant of the extent of surgical staging.

The present study has several limitations. Its retrospective design is susceptible to information and selection bias. The study included only 32 patients from a single tertiary care centre, limiting statistical power, precision, and generalizability. The cohort consisted of patients who underwent both preoperative MRI and definitive surgery, and therefore may not represent the broader population of patients with endometrial carcinoma. MRI interpretation was performed by a single experienced radiologist, and interobserver variability could not be assessed. Although the interval between MRI reporting and surgery was relatively short (three to 10 days, mean five days), minor changes in disease characteristics during this interval cannot be completely excluded. In addition, the small number of observations resulted in wide confidence intervals around the diagnostic performance estimates, and the findings should therefore be regarded as exploratory.

Despite these limitations, the study provides real-world data on MRI-histopathology concordance in endometrial carcinoma from a tertiary-care setting. The findings reinforce the usefulness of MRI as a preoperative imaging tool while highlighting the limitations of imaging-based assessment of borderline myometrial invasion. Larger prospective multicentre studies using standardized MRI protocols, structured radiological reporting, assessment by multiple radiologists, and integration of histopathological and molecular risk factors are required to better define the diagnostic and clinical utility of MRI in contemporary endometrial cancer management.

## Conclusions

In this small retrospective single-centre cohort, preoperative MRI demonstrated limited-to-moderate diagnostic performance in assessing the depth of myometrial invasion in endometrial carcinoma, with moderate overall accuracy and fair agreement with final histopathology. Although MRI provides valuable preoperative information for assessing local tumour extent and assisting surgical planning, discrepancies between MRI and histopathological findings were observed, particularly in cases of borderline or subtle myometrial invasion.

These findings suggest that MRI should be regarded as a complementary component of preoperative assessment rather than a substitute for definitive histopathological evaluation. The diagnostic performance estimates should be interpreted cautiously in view of the small sample size, retrospective design, single-centre setting, and inability to assess interobserver variability. Larger prospective multicentre studies using standardized MRI protocols and structured radiological assessment are required to validate these findings and determine the reproducibility and clinical utility of MRI in contemporary endometrial cancer staging.
